# EEMDS: An Effective Emergency Message Dissemination Scheme for Urban VANETs

**DOI:** 10.3390/s21051588

**Published:** 2021-02-25

**Authors:** Sami Ullah, Ghulam Abbas, Muhammad Waqas, Ziaul Haq Abbas, Shanshan Tu, Ibrahim A. Hameed

**Affiliations:** 1 Telecommunications and Networking Research Center, GIK Institute of Engineering Sciences and Technology, Topi 23640, Pakistan; sami@giki.edu.pk (S.U.); abbasg@giki.edu.pk (G.A.); engr.waqas2079@gmail.com (M.W.); ziaul.h.abbas@giki.edu.pk (Z.H.A.); 2 Faculty of Computer Science and Engineering, GIK Institute of Engineering Sciences and Technology, Topi 23640, Pakistan; 3 Faculty of Electrical Engineering, GIK Institute of Engineering Sciences and Technology, Topi 23640, Pakistan; 4 Engineering Research Center of Intelligent Perception and Autonomous Control, Faculty of Information Technology, Beijing University of Technology, Beijing 100124, China; sstu@bjut.edu.cn; 5 Faculty of Information Technology and Electrical Engineering, Norwegian University of Science and Technology, 7491 Trondheim, Norway

**Keywords:** road safety, vehicular adhoc networks, broadcast storms, clusters, communication congestion, flooding

## Abstract

In Vehicular Adhoc Networks (VANETs), disseminating Emergency Messages (EMs) to a maximum number of vehicles with low latency and low packet loss is critical for road safety. However, avoiding the broadcast storm and dealing with large-scale dissemination of EMs in urban VANETs, particularly at intersections, are the challenging tasks. The problems become even more challenging in a dense network. We propose an Effective Emergency Message Dissemination Scheme (EEMDS) for urban VANETs. The scheme is based on our mobility metrics to avoid communication overhead and to maintain a stable cluster structure. Every vehicle takes into account its direction angle and path loss factor for selecting a suitable cluster head. Moreover, we introduce estimated link stability to choose a suitable relay vehicle that reduces the number of rebroadcasts and communication congestion in the network. Simulation results show that EEMDS provides an acceptable end-to-end delay, information coverage, and packet delivery ratio compared to the eminent EM dissemination schemes.

## 1. Introduction

Safety applications in Vehicular Adhoc Networks (VANETs) mainly depend on disseminating Emergency Messages (EMs) [1]. Vehicles encountering a hazard disseminate EMs to other vehicles (hereinafter nodes) within their communication vicinity. This enables nodes to take adequate preventive measures, such as re-routing, to avoid road accidents, travel delays, and traffic congestion [2,3]. In VANETs, the most common and easiest way of disseminating EMs is flooding [4], in which a source node broadcasts EMs to other nodes within its transmission Range (*R*). In turn, the receiving nodes broadcast EMs in their *R* until the EMs propagate across the whole network. However, due to the dynamic nature of VANETs, flooding causes broadcast storms [5,6]. The consequent redundant transmission of EMs causes communication congestion, high delay, and degrades the message reliability.

For this reason, many methods, such as Store-Carry-Forward (SCF), and counter-based and distance-based disseminations have been proposed in the literature [7,8,9,10,11,12,13,14,15]. Nevertheless, SCF causes high End-to-End (E2E) delay while counter-based and distance-based methods are suitable only for well-connected networks. Moreover, without deploying a central coordinator unit, the threat of unnecessary retransmission may increase. Consequently, E2E delay and the packet loss rate can also increase, especially in high-density scenarios. These problems can be tackled by considering a cluster-based strategy that can establish a network hierarchy by organizing nodes based on certain predefined rules [16]. Each cluster has a coordinator unit, known as a Cluster Head (CH). Instead of rebroadcasting, the node in a cluster delivers the data to its CH for further dissemination. This strategy can effectively mitigate communication congestion and broadcast storms [17]. However, node clustering in VANETs has multiple open challenges, such as non-uniform node distribution, mobility, signal fading from neighboring nodes and other obstacles, as well as the overhead in cluster formation [18,19].

To address the aforementioned challenges, we propose a clustering-based Effective Emergency Message Dissemination Scheme (EEMDS) that considers our mobility metrics for the CH selection to increase cluster stability and to avoid communication overhead. Only the CH is responsible for disseminating EMs among its cluster members. Moreover, the estimated link-state stability (${L_{S}}_{T}$) metric for relay node selection suppresses retransmission of EMs across adjacent clusters and increases network efficiency. The novelty and contributions of this paper are as follows.

- We select CHs based on our mobility metrics, which include moving direction, velocity, distance, and time to leave. These metrics can increase the CH lifetime, reduce communication overhead and achieve a high Packet Delivery Ratio (PDR).
- We employ path loss factor using two-ray ground propagation model to consider both line-of-sight and the reflected signals for CH and relay node selection.
- We select a relay node, i.e., an intermediary that communicates among multiple clusters, by considering ${L_{S}}_{T}$ to overcome broadcast storms in high-density networks, and increases PDR with an acceptable delay.
- Each node takes into account its direction angle for selecting a suitable cluster. This is to avoid frequent switching of clusters and relay nodes at intersections in order to achieve high PDR.
- Simulation results show that EEMDS outperforms eminent EM dissemination schemes in terms of information coverage, PDR, and E2E delay.

The rest of the paper is organized as follows. Section 2 reviews the related work. Section 3 presents the system model. The proposed scheme and simulation results are presented in Section 4 and Section 5, respectively. Finally, Section 6 concludes the paper.

## 2. Related Work

The idea behind tackling communication congestion and the broadcast storm problem is to reduce redundant transmissions [20]. The existing approaches either permit only a limited number of nodes to retransmit EMs or restrict redundant EM transmissions. One of the existing approaches to permit a limited number of nodes to retransmit EM is a cluster-based approach. The authors in [21] propose a multi-hop cluster-based data dissemination scheme. The main criteria for CH selection are the relative distance and velocity to form a cluster. However, in dense networks, the traditional multi-hop broadcasting leads to high propagation delay, increased communication overhead, and low PDR. The authors in [22] devise a fog assisted data dissemination scheme. Every node updates its status, such as position, speed, and direction to the fog server. The server informs the connected nodes about emergent events and suggests adequate preventive measures. However, the scheme suffers high maintenance cost and communication delays. In [23], the authors propose an event-driven clustering to reduce communication congestion. Nevertheless, clustering after event identification leads to high propagation delay, and is suitable only for delay-tolerant information.

The authors in [24] propose a greedy routing scheme that considers link quality, segment node, and degree of link connectivity between communicating nodes to improve throughput and PDR. The scheme selects a region called segment area within the transmission range of a node. Nodes, which reside in the segment region, are called segment nodes. Thus, the choice of relay nodes depends on the quality of one-hop link, segment node, and degree of connectivity. In [25], the authors propose a Location Error Resilient Geographic Routing (LER-GR) scheme to improve the location accuracy of neighboring nodes. This scheme uses location prediction and error calculation to predict the location of single-hop neighbor nodes, which is then used as a relay node. To improve the reliability of the selected relay node and minimize communication congestion, [26] used particle swarm optimization to optimize the constraints related to the selection of a relay node, such as high interference, frequent topological changes, and limited forwarding direction. However, greedy routing is suitable only for well-connected networks, and swarm optimization has the limitations of impulsive and slow speed of convergence [27].

Similarly, in [28], the authors propose a position-based routing scheme for emergency message dissemination in VANETs. The scheme employes Geographic Information System (GIS) and electronic maps to create the spiderweb-like transmission model. Using GIS and electronic maps, the source node obtains its position, the destination node’s position, and the road layout. The source and destination confirm a route before message transmission by exchanging request-spiders and confirm-spiders packets. Hence, this model selects a stable path for EM transmission. However, due to high network overhead, this scheme cannot perform well in large-scale networks. In [29], the authors propose a position-based scheme for message routing on stable links. In this work, a link that remains active for a longer time is considered favorable for routing. Additionally, the work defines a recovery mechanism in a situation where the links break. However, the recovery strategies can create extra delay and communication overhead that deteriorate the network performance.

The authors in [30] present a Time Barrier-based Emergency Message Dissemination (TBEMD) scheme that integrates positional information with a time-barrier technique to minimize unnecessary EMs retransmissions. The most distant node within the source node’s *R* obtains the shortest back-off time. Hence, every node waits before rebroadcasting EMs. Nevertheless, the waiting time in the time-barrier technique leads to unnecessary delays in EM transmission. Moreover, there may be more than one node at the same distance. Thus, multiple nodes can transmit the same EM simultaneously, which adds to the communication congestion. The work in [31] presents a Distributed Vehicular Broadcast (DVCAST) technique to increase coverage using the Store-Carry-Forward (SCF) technique. However, the SCF technique incurs high delay. To minimize rebroadcasting, DVCAST employs inter-node distance to predict the probability that a particular receiver may become a relay node. In addition, to minimize the waiting time, a source node sends EMs to the farthest node with a high probability. Nevertheless, the probability of EM being sent increases exponentially as the distance increases. As a result, multiple nodes can retransmit EMs simultaneously and cause communication congestion.

Similarly, in [2], the network is hierarchically partitioned into several clusters on a highway, where all the cluster members are connected to a CH. In order to restrain redundant transmission for reliable EM dissemination, only CH is responsible for retransmitting EMs in each cluster. In addition, [2] uses a relay node to maximize coverage and has been shown to work well in highway environments. In this paper, we propose EEMDS as an extension of [2] to urban environments. Unlike [2], EEMDS employs path loss factor using two-ray ground propagation model to consider line-of-sight as well as the reflected signals for CH and relay node selection. Moreover, our mobility metrics and relay selection increase cluster stability and suppress retransmission of EMs. As a result, EEMDS provides high coverage to the nodes moving in the same direction with an acceptable delay and EM reliability. A comparison of various EM dissemination schemes is shown in Table 1.

## 3. System Model

This section describes the network model and the proposed mobility metrics (M) employed in EEMDS.

### 3.1. Network Model

**Figure 1:**
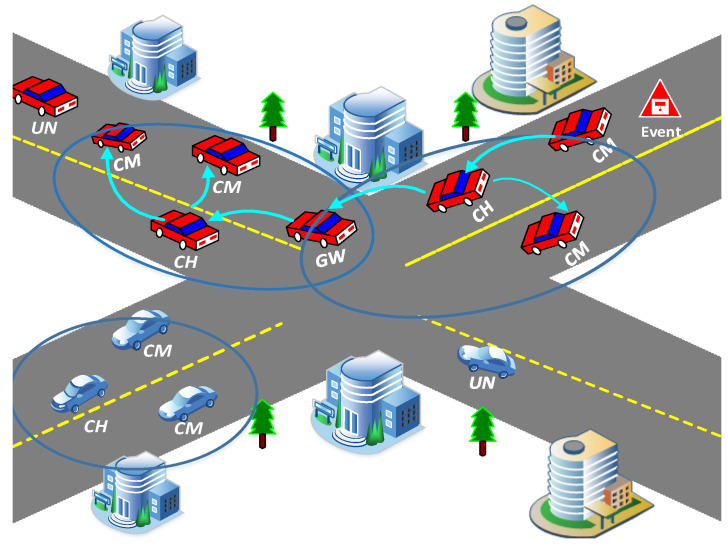
Network model.

The network consists of a set of nodes, *N*=$\left\{N_{1},N_{2},\cdots ,N_{n}\right\}$. Here, *n* shows the total number of nodes in the network. Moreover, we assume that each node is equipped with a Global Positioning System (GPS), and an Onboard Unit (OBU) that enables nodes to transmit beacons within their *R* to acquire necessary information, such as node position, node identifier (N_id), and speed. In EEMDS, nodes are categorized in one of the states mentioned below and depicted in Figure 1. Table 2 contains a list of notations used in the proposed scheme.

- Un-registered Node (UN): This is the initial state of a node. In this state, a node is not a member of any cluster (℧).
- Cluster Head (CH): A cluster head is responsible for coordinating with members of its cluster.
- Cluster Member (CM): Cluster members are the particular nodes in a cluster.
- Gateway (GW): A gateway acts as a relay node to provide connectivity between two clusters to extend information coverage without requiring road-side units [32].

### 3.2. Mobility Metrics

This subsection describes the considered mobility information in the proposed EEMDS. A single metric to select a node as a CH may reduce the network performance. Thus, the primacy of a node to become a CH relies on diverse M and neighborhood information, as listed below.

- Neighbor list (β): β depicts the set of neighbor nodes. Two nodes are called neighbors if they are within the transmission range of each other. Thus, β of node *i* can be computed as:
  (1) $$\beta_{i}=\left\{j|d_{i,j}<R\right\}.$$
  Here, *i*,*j*∈*N* and $d_{i,j}$ is the Euclidean distance between nodes *i* and *j*, which can be computed as [33]:
  (2) $$d_{i,j}=\sqrt{{\left(x_{i}-x_{j}\right)}^{2}+{\left(y_{i}-y_{j}\right)}^{2}},$$
  where $\left(x_{i},y_{i}\right)$ and $\left(x_{j},y_{j}\right)$ are the X, Y coordinates of nodes *i* and *j*, respectively.
- Cardinality (γ): γ is the number of nodes in set β. Thus, γ of node *i* can be calculated as [2]:
  (3) $$\gamma_{i}=\left|\beta_{i}\right|.$$
- Moving direction: Nodes in the same direction will maintain a relatively stable connection with their respective CH. Therefore, we cluster nodes according to their movement direction. Hence, nodes *i* and *j* will be in the similar direction if θ ≤π/4, where θ is the angle between the velocity vector of nodes *i* and *j*. Let the position (X, Y coordinate) of nodes *i* and *j* be $\left(x_{i},y_{i}\right)$, $\left(x_{j},y_{j}\right)$ and ($\bar{x_{i}}$, $\bar{y_{i}}$), ($\bar{x_{j}}$, $\bar{y_{j}}$) at time step $t_{1}$ and $t_{2}$, respectively, then angle between reference nodes *i* and *j* can be expressed as [34]:
  (4) $$\theta_{i,j}=\arccos\left(\frac{\delta x_{i}\delta x_{j}+\delta y_{i}\delta y_{j}}{\sqrt{\delta x_{i}^{2}+\delta y_{i}^{2}}\sqrt{\delta x_{j}^{2}+\delta y_{j}^{2}}}\right).$$
  Here, δx and δy show the change in position of nodes *i* and *j*, respectively, in time interval *t*.
- Normalized Average Relative Distance (RD): A node with minimum RD is closer to the center of its β. Therefore, the node having minimum RD will be a potential candidate for CH. Hence, we can express RD of node *i* relative to *j*, such that $\forall j\in \beta_{i}$, as:
  (5) $${\mathrm{RD}}_{i}=\frac{1}{\gamma_{i}}\;\frac{\sum_{j=1}^{\gamma_{i}}d_{i,j}}{\max\{d_{i,j}\}};\;j\neq i.$$
- Normalized Average Relative Velocity (RV): A node with lower RV as compared to other nodes in its β entails that it has a more stable state. This implies that the node will stay for a longer duration in its own cluster area as compared to other nodes. Suppose $v_{i}$ and $v_{j}$ are the velocities of nodes *i* and *j*, respectively, then we can compute RV as:
  (6) $${\mathrm{RV}}_{i}=\frac{1}{\gamma_{i}}\;\frac{\sum_{j=1}^{\gamma_{i}}\left|v_{i}-v_{j}\right|}{\max\{|v_{i}-v_{j}|\}};\;\forall j\in \beta_{i},\;j\neq i.$$
- Normalized Average Path Loss (NP): Path Loss (PL) shows the impact of fading on signals. A node having lower PL value with respect to other nodes is likely to become a CH. The relative PL between nodes *i* and *j* can be calculated as [35]:
  (7) $${\mathrm{PL}}_{i,j}\left[\mathrm{dB}\right]=10\log_{10}\left(16\pi^{2}\frac{d_{i,j}^{\alpha }}{\lambda^{\alpha }}\right),$$
  where $d_{i,j}$ is the Euclidean distance between nodes *i* and *j*. Here, λ shows the wavelength and α is environment-dependent path loss exponent given in Table 3. Hence, NP of node *i* as compared to *j*, such that $\forall j\in \beta_{i}$, can be computed as:
  (8) $${\mathrm{NP}}_{i}\left[\mathrm{dB}\right]=\frac{1}{\gamma_{i}}\;\frac{\sum_{j=1}^{\gamma_{i}}PL_{i,j}}{\max\{PL_{i,j}\}}\;;j\neq i.$$
- Time to Leave (TL): Each node periodically computes TL for leaving the road segment based on its present GPS location. A node having a long-lasting TL can increase the cluster stability. Thus, TL of node *i* can be computed as [36]:
  (9) $${\mathrm{TL}}_{i}=\frac{L-D_{i}}{D_{i}},$$
  where $D_{i}$ is node *i*’s covered distance in time interval *t* and *L* is the road-segment length.

Thus, we can express M of node *i* based on (5), (6), (8) and (9), respectively, as:

(10) $$M_{i}={\mathrm{RD}}_{i}+{\mathrm{RV}}_{i}+{\mathrm{NP}}_{i}-{\mathrm{TL}}_{i}.$$

Hence, a node with a lower M value will be selected as a CH.

## 4. The Proposed Scheme

In EEMDS, nodes are organized in clusters as depicted in Figure 1. In every cluster, CH is responsible for managing CMs and controlling EM dissemination. The value of M is calculated according to (10) for the sake of cluster stability and CH selection. In addition, we define the link stability metric in this section for GW selection to limit the number of nodes for EM retransmision across the cluster. EEMDS consists of the following phases.

### 4.1. Neighbor Discovery Phase

Every node periodically broadcasts beacon messages to the neighbor nodes to exchange information, such as N_id, velocity, position, and node state. The receiving node updates its β based on (2) and (4), which can be used in a cluster formation phase.

We present Algorithm 1 for the the neighborhood discovery of node *i*. Algorithm 1 takes *N* as input and produces $\beta_{i}$ as output. Upon receiving beacon from any node *j*, j∈N, node *i* uses (2) and (4) to confirm node *j* eligibility as a valid neighbor. After confirmation, node *i* adds node *j* to its $\beta_{i}$.
**Algorithm 1:**Neighbor discovery.
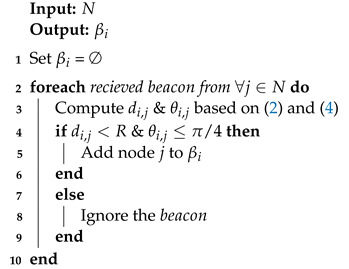


### 4.2. Cluster Formation Phase

When a UN node wants to create or join a cluster, it broadcasts a beacon to other nodes. The beacon contains the node’s state, velocity, and position information. Similarly, each CH also broadcasts a Cluster Head Advertisement (CHA) message containing its velocity, M, Cluster id (C_id), and position. Consequently, when node *i* receives a beacon or CHA message, it uses (4) to determine its direction relative to the corresponding sender’s direction. Upon updating β, node *i* calculates its M value based on (10) and exchanges it with neighbor nodes in $\beta_{i}$. When only one CH exists in node *i*’s β, it sends a Request to Join Cluster (RJC) message to the CH containing its N_id and becomes a CM.

Whenever node *i*’s β contains more than one CHs, it selects a CH that has the lowest M value and sends an RJC to the selected CH. Unless node *i*’s β does not contain a CH, it compares its M with all the neighbor nodes. If the M value of node *i* is smaller than that of any other node in its β, it announces itself as the CH. Hence, the newly selected CH will announce CHA to nodes in $\beta_{i}$, which contains its $M_{i}$ value and C_id. Upon receiving CHA, if $M_{i}$ is the lowest as compared to those received from all other CHs, the other nodes in $\beta_{i}$ will respond by sending an RJC to node *i*. After receiving RJC from any node *j*, such that j∈$\beta_{i}$, node *i* will record node *j*’s N_id in its CMT. Hence, node *j* will become a CM. Contrarily, node *j* will record N_id of node *i* in its Cluster Head Table (CHT). Algorithm 2 presents the complete procedure of cluster formation in EEMDS.
**Algorithm 2:**Cluster formation.
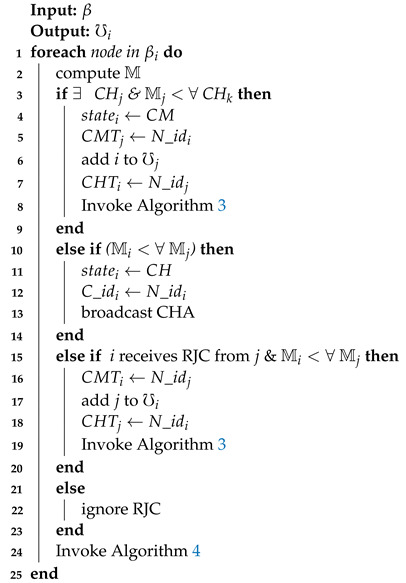


### 4.3. Gateway Selection Phase

A CH selects two CMs, which travel on the cluster boundary, to be a potential GW. To that end, ${L_{S}}_{T}$ is taken into account between the CH and the CMs, which can be calculated as:

(11) $${L_{S}}_{T}i,j=\frac{{\mathrm{RV}}_{i,j}}{{\mathrm{RD}}_{i,j}}R,$$

where ${\mathrm{RD}}_{i,j}$ and ${\mathrm{RV}}_{i,j}$ are the relative average distance and velocity, between nodes *i* and *j*, based on (5) and (6), respectively. A node with a lower ${L_{S}}_{T}$ value shows a more stable connection and is selected as GW. Algorithm 3 demonstrates the procedure of GW selection.

### 4.4. Cluster Maintenance Phase

VANETs are highly dynamic in nature due to high-speed mobility of nodes and frequent topological changes. Nodes usually join and leave clusters frequently that causes link disconnection between CMs and CH, resulting in a high packet loss ratio. To decrease the packet loss ratio due to link disconnection between *CM*s and a CH, a cluster should be maintained regularly. Hence, in EEMDS, once a cluster is created, each CM periodically broadcasts Cluster Member Advertisement (CMA) packets to demonstrate its presence in the network. Similarly, CH broadcasts CHA packets. In this way, CM and CH identify the presence of each other and maintain the cluster structure as shown in Algorithm 4. If a CM loses contact with its respective CH, it updates its state according to Algorithm 4. Similarly, if a CH cannot hear CMA and loses contact with its CMs, the CH updates its CMT. The CH also changes its state when no more CMs exist in its CMT.


**Algorithm 3:**Gateway selection.

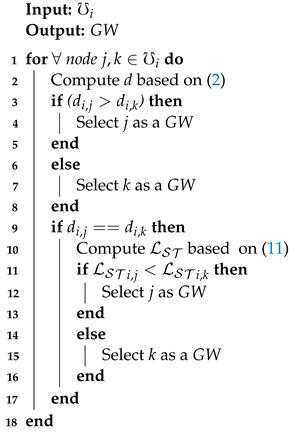





**Algorithm 4:**Cluster maintenance.

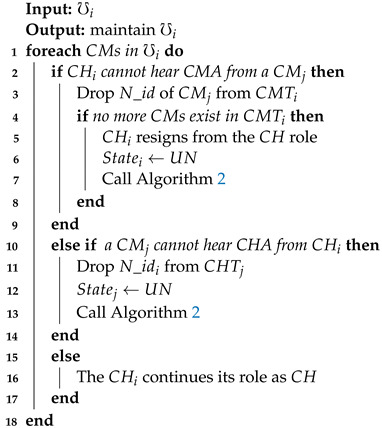




### 4.5. Emergency Message Dissemination Phase

**Figure 2:**
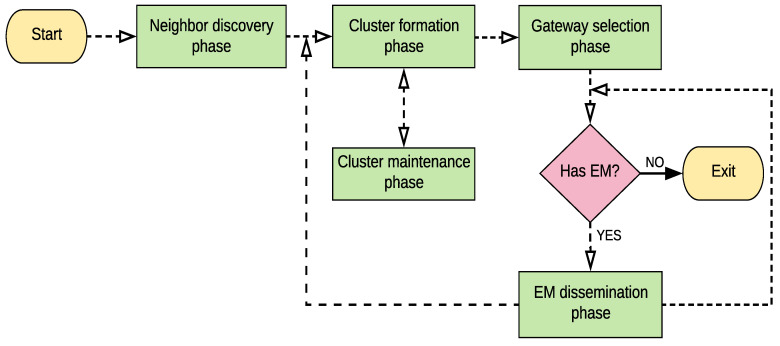
Procedural flowchart of EEMDS.

EEMDS aims to increase the efficiency of EMs dissemination in VANETs. In conventional techniques, EMs are broadcasted, which leads to communication congestion and results in high packet loss ratio and E2E delay. In EEMDS, CH is responsible for disseminating EMs to its CMs. When the receiver is a CM, it sends EM to the corresponding CH for further dissemination. To expand the coverage area, EEMDS uses GW to disseminate EMs to the neighboring clusters. To prevent multiple nodes from sending the same EM, a GW based on (11) is used to disseminate EMs. As a result, ${L_{S}}_{T}$ enables EEMDS to tackle broadcast storms and expands the coverage area. The process of EM dissemination is described in Algorithm 5. Figure 2 presents the procedural flowchart of EEMDS.
**Algorithm 5:**Emergency message dissemination.
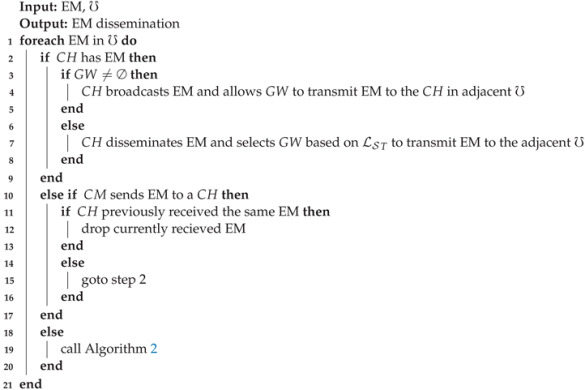


## 5. Performance Evaluation

We now present performance evaluation of the proposed EEMDS in comparison with flooding [4], TBEMD [30], and DVCAST [31]. To evaluate performance in a realistic vehicular environment, we use Mobility Model Generator for Vehicular Networks (MOVE) [37], Simulation of Urban Mobility (SUMO) [38], and ns-2.35. MOVE and SUMO enable users to generate real-world mobility models for VANETs simulations. MOVE works in integration with the open-source micro-traffic simulator SUMO. The output of SUMO and MOVE consists of node positions, intersections, and route information, which is used by ns-2. Mobility is evaluated on the urban road with two lanes, according to the Krauss mobility model [39]. We consider 300 m distance as the *R* and node density 25/km to 150/km. Table 4 shows the parameters used in the simulations. Performance metrics include information coverage, packet delivery ratio, E2E delay, and cluster stability.

### 5.1. Information Coverage

**Figure 3:**
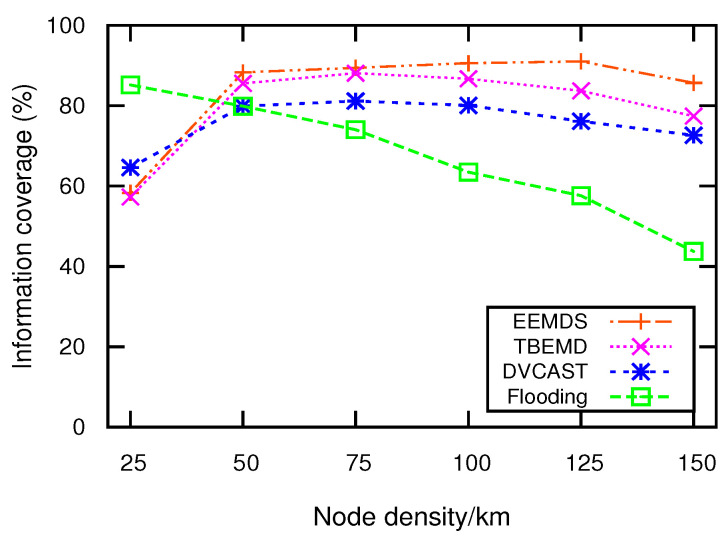
Information coverage vs. node density.

Information coverage is the percentage of nodes in the network that successfully receive EM. Figure 3 illustrates information coverage relative to node density. In low-density networks, traditional flooding outperforms TBEMD, DVCAST, and EEMDS, respectively. This is because, in flooding, every node rebroadcasts the message without any restrictions. However, excessive rebroadcasts lead to the storms in high-density networks, causing communication congestion and reduced information coverage. TBEMD, DVCAST, and EEMDS show similar performance in low-density. However, when density becomes high, EEMDS outperforms TBEMD, DVCAST, and flooding. The reason is that EEMDS reaches a higher number of close neighbors due to its stable clustering structure and also controls unnecessary retransmissions by using its unique relay node selection strategy. Conversely, TBEMD and DVCAST have low information coverage due to fewer close neighbors and a high number of retransmissions. We observe that EEMDS increases the average information coverage by 8%, 13.2%, and 20.7%, compared to TBEMD, DVCAST, and flooding, respectively.

### 5.2. E2E Delay

**Figure 4:**
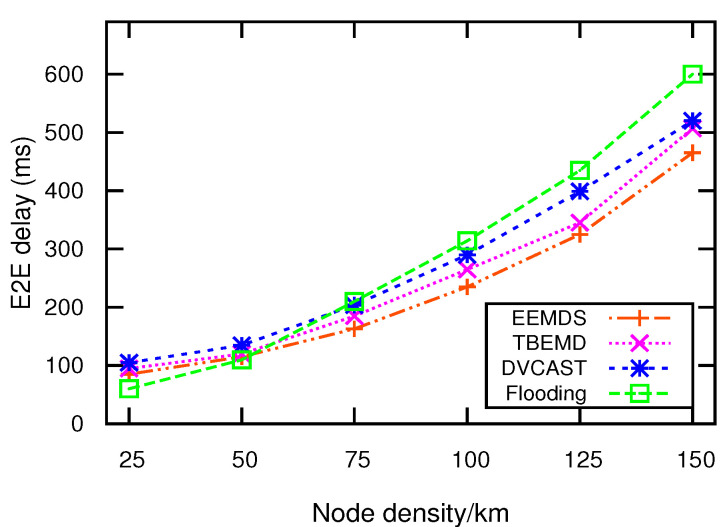
E2E delay vs. node density.

E2E delay is the time taken for an EM to traverse from a source to destination. Figure 4 shows the impact of node density on E2E delay. Traditional flooding outperforms TBEMD, DVCAST, and EEMDS in low node density. However, flooding generates a large number of redundant transmissions in a high-density environment. Consequently, it causes communication congestion and produces higher E2E delay. DVCAST employs the SCF technique to maximize coverage and distance-based probabilistic technique for the selection of a relay node. However, SCF causes high delay. Moreover, in distance-based probabilistic technique, multiple nodes can send EMs simultaneously with the same probability, which leads to communication congestion. Consequently, DVCAST produces high E2E delay. In TBEMD, the nodes are allowed to retransmit EMs when their time barriers become out-dated. This retransmission increases communication congestion, particularly in a high-density network, resulting in a higher E2E delay. Contrarily, EEMDS uses its ${L_{S}}_{T}$ metric for reliable relay selection, which prevents multiple nodes from concurrent EM transmissions. Consequently, EEMDS overcomes excessive communication congestion and decreases the average E2E delay by 12%, 11.26%, and 20.08%, as compared to TBEMD, flooding, and DVCAST, respectively.

### 5.3. Packet Delivery Ratio

**Figure 5:**
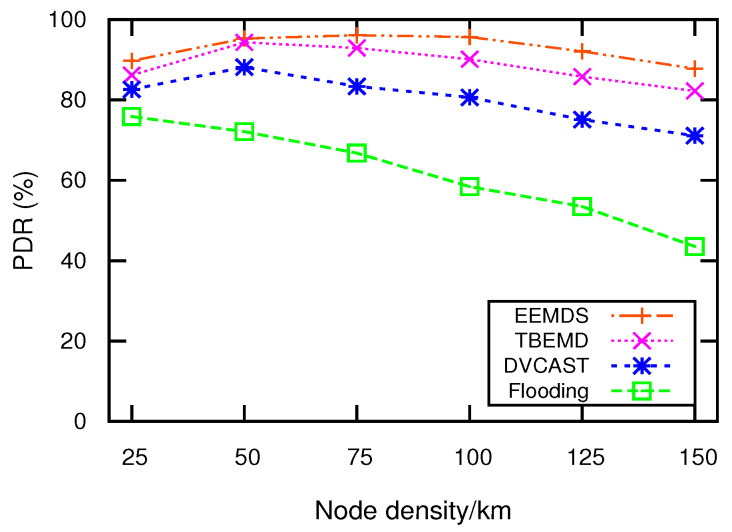
PDR vs. node density.

PDR is the ratio of the number of packets successfully delivered to the destination and the number of packets transmitted by the source. Figure 5 depicts PDR relative to node density. It can be observed that the increasing density has a positive impact on the performance of TBEMD, DVCAST, and EEMDS. The reason is that when the number of nodes increases, the network connectivity increases, which increases the successful delivery of the packets among the nodes. However, as the network becomes denser, the transmission of packets increases, which results in higher congestion and packet drops. Figure 5 illustrates that EEMDS outperforms flooding, TBEMD, and DVCAST. The reason is that DVCAST and TBEMD select relay nodes based on distance without considering other necessary parameters, such as velocity and link stability. Selecting relay node solely on distance can make the nodes to rebroadcast EM simultaneously, which increases communication congestion and degrades PDR. Contrarily, EEMDS suppresses concurrent EM broadcasting due to its reliable relay, which can play a significant role during rush hours in the real networks. As an example, for 125 nodes/km, we observe that EEMDS has 7.39%, 22.69%, and 62.28% more PDR compared to TBEMD, DVCAST, and flooding, respectively.

**Figure 6:**
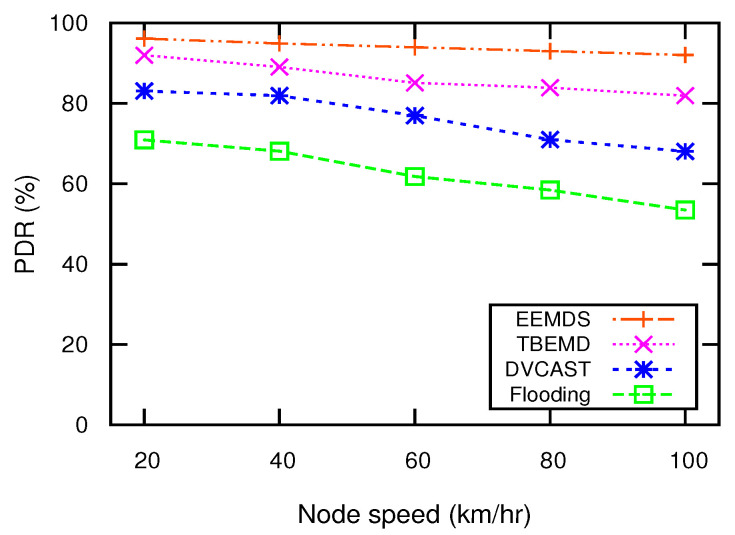
PDR vs. node speed.

Figure 6 illustrates the impact of nodes’ speed on PDR, where the node density is set to 75/km. The increase in speed leads to rapid changes in the network topology, which effects the PDR. For DVCAST and TBEMD, the farthest node has a higher priority to forward EM. However, selecting the farthest node based solely on distance without considering the mobility can lead to an unstable cluster, which increases communication congestion and degrades PDR. Hence, the frequent topological changes due to high mobility degrade PDR in DVCAST, TBEMD, and flooding. From Figure 6, it can be observed that EEMDS outperforms DVCAST, TBEMD, and flooding schemes. This is because EEMDS gives high priority to the cluster stability and node mobility to form a stable network structure. A stable network structure enables the nodes in EEMDS to communicate for longer time to maintain high PDR. Therefore, its PDR just slightly decreases with the increasing speed of nodes. To sum up, EEMDS demonstrates an average increase in PDR by 8.9%, 23.97%, and 43.07% compared to DVCAST, TBEMD, and flooding, respectively.

### 5.4. Cluster Stability

**Figure 7:**
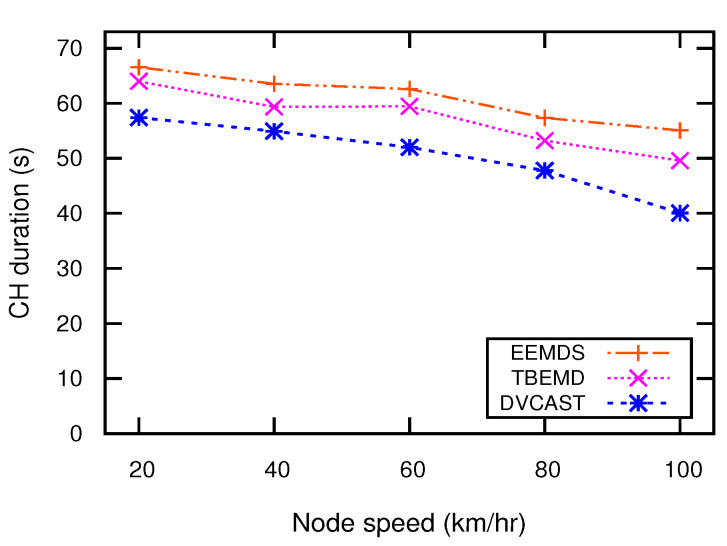
CH duration vs. node speed.

Cluster stability means that the cluster configuration should not change drastically while the topology changes. For effective EM dissemination, the clustering scheme must be stable because an unstable cluster structure increases network load that degrades the network performance. To maintain cluster stability, a good clustering algorithm has high CHs and CMs duration. Figure 7 illustrates the CH duration of EEMDS, TBEMD, and DVCAST for varying nodes’ speed. CH duration refers to the interval during which the nodes’ state is in CH and remains in this state until its state changes to UN or CM. A high duration of CH shows stable cluster structure. The results in Figure 7 show that CH duration decreases when the speed of nodes increase. This is because when the nodes’ speed increase, the network topology becomes more dynamic. Consequently, CHs cannot maintain a relatively stable state with their CMs for a long duration. From Figure 7, it can be observed that EEMDS obtains the longest CH duration as compared to DVCAST and TBEMD. The reason is that EEMDS employs the mobility metrics to select a stable CH that eventually enables EEMDS to sustain its state and maintain a long-lasting connection with its CMs.

**Figure 8:**
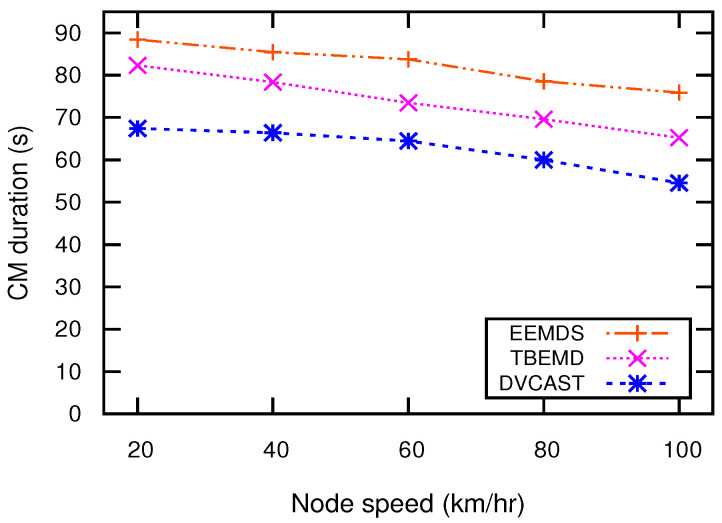
CM duration vs. node speed.

Figure 8 shows the CM duration of EEMDS, TBEMD, and DVCAST at different permissible speeds. CM duration is the time interval when a node joins a specific cluster until it leaves the cluster or changes its state. Figure 8 illustrates that the CMs duration decreases with the increase in speed. The reason is that, due to high speed, it is difficult for CMs and CHs to maintain a connection with each other for a long duration. However, the similar driving directions and the selection of a stable CH, EEMDS acquires a high CM duration as compared to DVCAST and TBEMD.

### 5.5. Impact of Transmission Range on EEMDS

**Figure 9:**
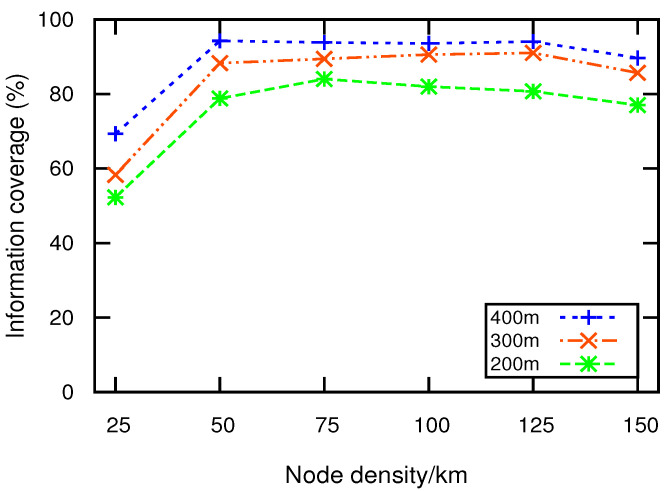
Information coverage vs. node density.

**Figure 10:**
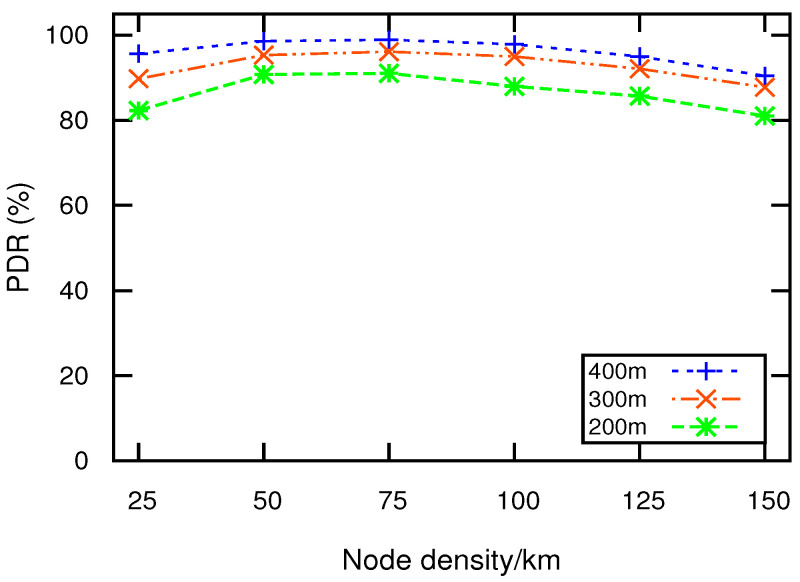
PDR vs. node density.

Figure 9 and Figure 10 illustrate the performance of EEMDS as functions of node densities and transmission range *R*. It can be observed from the figures that the increase in *R* has a positive effect on the EEMDS performance. Figure 9 illustrates that the information coverage is improved with the extended *R*. This is due to the fact that a higher *R* increases the number of neighbors by covering a larger region with stronger signal strength. A similar impact has been noticed in PDR, as shown in Figure 10. This is because extended *R* produces high connectivity among nodes in a sparse network.

### 5.6. Critical Discussion

Accident prevention through EM dissemination is one of the most significant services provided by VANETs. However, the unpredictable behavior of VANETs with rapid topological changes, high mobility, and short communication range of wireless nodes make it challenging to develop an effective EM dissemination scheme that provides low E2E delay, high PDR, and extended information coverage. In order to achieve extended information coverage and low E2E delay in EM dissemination, a widely used approach is broadcasting by flooding. Nevertheless, extensive broadcasting leads to communication congestion that degrades the network performance. To tackle this problem, several research studies select the farthest node to rebroadcast EMs. However, selecting the farthest node based solely on distance without taking into account other important parameters, such as velocity, transmission range, and link stability can make the nodes to rebroadcast EMs concurrently. The concurrent rebroadcasting increases communication congestion, which impedes the timely delivery of EMs to effectively prevent accidents. To address these issues, we have proposed a cluster-based EM dissemination scheme, called EEMDS, which is based on our mobility metrics to build a stable cluster to reduce the overhead of cluster formation and, thereby, increase EM reliability. EEMDS selects gateways based on ${L_{S}}_{T}$ to prevent multiple nodes from disseminating EMs concurrently and to gain extended information coverage.

Simulation results presented in the previous subsections demonstrate the robustness of EEMDS in addressing the aforementioned challenges to a reasonable extent. Performance evaluation reveals that, in contrast to the benchmark schemes, EEMDS performs reasonably well in terms of the considered network performance parameters, including PDR, E2E delay, and information coverage. As timely delivery of safety messages is massively crucial, reducing E2E delay is, therefore, valuable. EEMDS has also been shown to increase information coverage and PDR. This is enabled by the use of the ${L_{S}}_{T}$ metric, which helps to prevent multiple nodes from concurrently rebroadcasting the same EM and suppress excessive retransmission and communication congestion in dense urban networks. Contrarily, multiple nodes rebroadcast the same EM in the benchmark schemes, which causes communication congestion and results in performance degradation in high-density urban VANETs. The results reveal reduced E2E delay for EEMDS by 12%, 20.08%, 11.26%, as compared to TBEMD, DVCAST, and flooding, respectively. Considering average information coverage and PDR, EEMDS has improved information coverage by 8%, 13.2%, and 20.7%, and PDR by 9%, 20%, and 51%, as compared to TBEMD, DVCAST, and flooding, respectively.

The improved performance and robustness of EEMDS increase the efficiency of urban VANETs in the dissemination of emergency messages to enable vehicles to take preventive measures beforehand to avoid road accidents. Nevertheless, even though EEMDS has shown to improve network efficiency reasonably in high-density urban networks, the limited transmission range in vehicle-to-vehicle communication model can decrease its performance in sparse networks. Our future work will seek to tackle this limitation.

## 6. Conclusions

This paper has proposed an EM dissemination scheme, called EEMDS, to overcome unnecessary retransmissions and achieve high information coverage, PDR, and low E2E delay in urban VANETs. A clustering scheme based on mobility metrics has been presented to select a suitable CH and form stable clusters. Moreover, we have proposed link stability metric to select a reliable relay node to limit the number of nodes for inter-cluster communication. The link stability metric and stable cluster structure in EEMDS enable EM dissemination to a large number of nodes with acceptable delay. Simulation results reveal that EEMDS outperforms eminent existing schemes in terms of information coverage, PDR, and E2E delay.

## Figures and Tables

**Table 1:** Comparison of VANET EM dissemination schemes.

Scheme	Network Scenario	Relay Selection Parameters	EM Dissemination Mechanism	PDR	Delay	Node Density
ine DVCAST [31]	Highway	Distance	Broadcast, SCF	Medium	High	High
ine Ullah et al. [2]	Highway	Distance, ${L_{S}}_{T}$	Broadcast, uni-cast	High	Low	High
ine Schwartz et al. [10]	Highway	Distance, Direction	Broadcast, SCF	High	Medium	Medium
ine Chen et al. [11]	Highway	Distance	Broadcast, SCF	Medium	Medium	Medium
ine Nguyen et al. [14]	Highway	Random	SCF	Medium	Medium	High
ine Kamakshi et al. [15]	Highway	Dominating set	Broadcast	Medium	Medium	High
ine Flooding [4]	Urban	Pure flooding, behind node	Broadcast	Low	Medium	Low
ine Viriyasitavat et al. [12]	Urban	Distance, angle	Broadcast, SCF	High	Medium	High
ine TBEMD [30]	Urban	Distance	Broadcast	Medium	Medium	High
ine Yaqoob et al. [22]	Urban	Distance	FoG server-based	Medium	Medium	Low
ine Benkerdagh et al. [23]	Urban	Fitness function	Broadcast	Medium	High	High
ine Pal et al. [13]	Urban	Distance	Broadcast	Medium	Medium	Low
ine Qiu et al. [28]	Urban	Delay-based	Uni-cast, SCF	High	Low	High
ine EEMDS	Urban	Distance, link stability	Broadcast, uni-cast	High	Low	High

**Table 2:** List of notations.

Symbols	Description
CH, CM	Cluster head and cluster member, respectively
*R*, ℧	Transmission range and cluster, respectively
UN, $L_{S}T$	Un-registered node and estimated link stability, respectively
CHT, CMT	Cluster head table and cluster member table, respectively
N_id, C_id	Node id and cluster id, respectively
$M_{i}$, $\beta_{i}$	Mobility metrics and neighbor list of node *i*, respectively
$\gamma_{i}$, ${\mathrm{TL}}_{i}$	Cardinality of $\beta_{i}$ and time to leave of node *i*, respectively
$d_{i,j}$	Euclidean distance between nodes *i* and *j*
${\mathrm{NP}}_{i}$	Relative average path loss of node *i*
${\mathrm{RV}}_{i}$	Relative average velocity of node *i*
${\mathrm{RD}}_{i}$	Relative average distance of node *i*
${\mathrm{PL}}_{i,j}$	Relative path loss of nodes *i* and *j*

**Table 3:** Path loss exponent values.

Environment	Path Loss Exponent, α
Indoor	1.6–1.8
Suburban area	3.0–5.0
Urban area	2.7–3.5
Free space	2.0–4.43

**Table 4:** Simulation Parameters.

Parameters	Values
Propagation model	Two-ray ground
Mobility model	Krauss
Wireless access	Wave I609/802.11p
Transmission range	300 m
Transmission power	20 mW
Frequency	5.9 GHz
Simulation area	4000 m × 4000 m
Simulation time	500 s
Data rate, EM size	6 Mbps, 170 Bytes, respectively
Node speed	20–100 km/h
Node density	25–150/km
Number of lanes	2
beacon periodic interval	150 ms

## Data Availability

Not applicable.
